# Preoperative neutrophil-to-lymphocyte ratio is associated with amputation level progression in patients with diabetic foot

**DOI:** 10.3389/fsurg.2026.1868923

**Published:** 2026-08-21

**Authors:** Ismail Tugay Yagci, Ebru Alanbay Yagci, Ali Baran Budak, Osman Tugrul Eren

**Affiliations:** 1 Department of Orthopaedics and Traumatology, Istinye Universitesi Tip Fakultesi, İstanbul, Türkiye; 2 Department of Orthopedics and Traumatology, Istinye University, Liv Hospital Ulus, Istanbul, Türkiye; 3 Division of Pain Medicine, Department of Physical Medicine and Rehabilitation, Muş State Hospital, Muş, Türkiye; 4 Department of Cardiovascular Surgery, Istanbul Aydın University Faculty of Medicine, Istanbul, Türkiye; 5 Department of Orthopedics and Traumatology, University of Health Sciences, Şişli Hamidiye Etfal Training and Research Hospital, Istanbul, Türkiye

**Keywords:** above-knee amputation, below-knee amputation, diabetic foot, neutrophil-to-lymphocyte ratio (NLR), systemic inflammation

## Abstract

**Background:**

Amputation-level progression after below-knee amputation (BKA) is associated with functional and psychological burden in diabetic foot disease. We evaluated the association between preoperative neutrophil-to-lymphocyte ratio (NLR) and short-term progression from BKA to above-knee amputation (AKA).

**Methods:**

This single-center retrospective cohort included 105 consecutive adults undergoing primary transtibial BKA for diabetic foot complications. The primary outcome was conversion from the index BKA to AKA within 3 months. Demographic, clinical, laboratory, disease severity, perioperative, and 12-Item Short Form Health Survey Version 1 (SF-12v1) data were obtained from medical records. A parsimonious multivariable logistic regression model including age, Wound, Ischemia, and foot Infection (WIfI) stage, Wagner grade, C-reactive protein (CRP), serum albumin, and NLR evaluated the adjusted association between NLR and amputation-level progression. Sensitivity analyses examined NLR estimate stability across alternative specifications. Receiver operating characteristic analysis explored the apparent discriminative ability of NLR.

**Results:**

Within 3 months, 53 of 105 patients (50.5%) progressed from BKA to AKA. Median preoperative NLR was higher among patients with progression than among those maintaining the initial BKA level [10.42 (interquartile range: 5.68–13.03) vs. 5.47 (3.24–9.04), *p* < 0.001]. Patients with progression also had higher neutrophil and lower lymphocyte counts (both *p* = 0.006). In the primary model, each one-unit increase in NLR was associated with 29% higher odds of progression (adjusted odds ratio: 1.29, 95% confidence interval: 1.14–1.47, *p* < 0.001). The association remained significant after excluding WIfI stage, CRP, Wagner grade, or both CRP and Wagner grade. Exploratory ROC analysis yielded an apparent area under the curve of 0.73. Postoperative SF-12v1 mental component scores, assessed approximately 3 weeks after BKA and before AKA conversion, were lower among patients with progression (*p* < 0.001).

**Conclusions:**

Preoperative NLR was associated with early amputation-level progression after adjustment for available covariates. NLR may provide adjunctive information regarding systemic inflammatory burden but should not be interpreted as a validated standalone predictor or clinical decision threshold. These hypothesis-generating findings require confirmation in larger prospective multicenter cohorts with comprehensive vascular data and internal and external validation.

## 1 Introduction

Diabetic foot disease is one of the most devastating complications of diabetes mellitus and remains a leading cause of non-traumatic lower-extremity amputation worldwide. It is estimated that up to 25% of patients with diabetes will develop a foot ulcer during their lifetime, and diabetic foot complications account for a substantial proportion of diabetes-related hospitalizations and amputations (1, 2). Despite advances in multidisciplinary care, including improved infection management, vascular interventions, and modern wound care strategies, major amputations continue to occur in patients with advanced infection, ischemia, and tissue necrosis (3).

Diabetic foot disease and major lower-extremity amputation are associated with profound physical, psychological, and socioeconomic consequences. Affected patients may experience reduced mobility and independence, prolonged rehabilitation needs, and impaired health-related quality of life (4). Diabetic foot complications also impose a considerable burden on healthcare systems due to prolonged hospital stays, repeated surgical interventions, wound care requirements, and long-term rehabilitation. Therefore, identifying patients at risk for poor limb-related outcomes, including amputation-level progression, remains an important clinical challenge in diabetic foot management.

Several clinical classification systems have been developed to evaluate disease severity and guide treatment strategies in patients with diabetic foot disease. The Wagner classification system, which categorizes ulcers according to depth and the presence of gangrene, has historically been one of the most widely used grading systems (5). More recently, the Wound, Ischemia, and foot Infection (WIfI) classification system introduced by the Society for Vascular Surgery has been proposed as a comprehensive tool for estimating limb threat by integrating wound characteristics, ischemia severity, and infection status (6). Although these systems provide valuable information regarding local disease severity, they may not fully capture the systemic inflammatory response associated with advanced diabetic foot infection and tissue loss.

Systemic inflammatory markers have gained increasing attention as potential adjunctive clinical markers in diabetic foot disease. Among these markers, the neutrophil-to-lymphocyte ratio (NLR) is a simple, inexpensive, and readily available index derived from routine complete blood count parameters. NLR reflects the balance between neutrophil-mediated inflammatory activation and lymphocyte-mediated immune regulation and has been associated with adverse outcomes in several clinical settings, including infection severity, cardiovascular events, and surgical outcomes (7, 8).

Previous studies have demonstrated that elevated NLR is associated with increased diabetic foot infection severity and a higher risk of major amputation (9, 10). However, most available studies have focused on the occurrence of amputation or limb loss in general. Less is known about whether preoperative NLR is associated with short-term amputation-level progression among patients who have already undergone below-knee amputation (BKA). This is a clinically relevant endpoint because progression from BKA to above-knee amputation (AKA) may substantially affect mobility, rehabilitation potential, prosthetic use, and psychological outcomes.

Therefore, the aim of the present study was to evaluate the association between preoperative NLR and short-term amputation-level progression from BKA to AKA in patients with diabetic foot disease. In addition, early postoperative health-related quality-of-life outcomes were examined in this patient cohort.

## 2 Methods

### 2.1 Study design and participants

This retrospective cohort study included consecutive adult patients who underwent primary transtibial below-knee amputation (BKA) for diabetic foot complications between January 2020 and December 2022. A total of 144 patients were screened for eligibility. Thirty-nine patients were excluded because of a previous major lower-extremity amputation (*n* = 12), traumatic or malignancy-related amputation (*n* = 8), incomplete medical records (*n* = 7), insufficient 3-month follow-up data (*n* = 11), or death before 3-month outcome ascertainment without documented progression to above-knee amputation (AKA) (*n* = 1). The final analytic cohort comprised 105 patients. At 3 months, 52 patients had maintained the initial BKA level, whereas 53 had undergone progression from BKA to AKA. The patient selection process is presented in Supplementary Figure S1.

### 2.2 Patient selection

Patients older than 18 years who underwent primary transtibial below-knee amputation (BKA) for diabetic foot complications were included. In this study, BKA was defined as transtibial major amputation performed between the knee and ankle joints with preservation of the knee joint. Below-ankle amputations, ankle disarticulations, Syme amputations, heel-sparing procedures, ray amputations, and transmetatarsal amputations were not included. Above-knee amputation (AKA) was defined as transfemoral major amputation.

Patients with previous major lower-extremity amputation, traumatic or malignancy-related amputation, incomplete medical records, insufficient 3-month follow-up data, or death before 3-month outcome ascertainment without documented progression to AKA were excluded.

### 2.3 Outcome definition

The primary outcome was early amputation-level progression, defined as conversion from the index below-knee amputation (BKA) to above-knee amputation (AKA) within 3 months of the initial procedure. The 3-month period was selected *a priori* to capture short-term postoperative progression potentially reflecting early stump failure, persistent or progressive infection, ischemic tissue loss, or inadequate local disease control after the index amputation.

Conversion to AKA was a clinical treatment decision made by the treating orthopedic surgical team, with consultation from vascular surgery and infectious disease specialists when clinically indicated. The decision was based on the treating team's documented assessment that preservation of a viable and functional transtibial residual limb was no longer feasible. Recorded indications included one or more of the following: progressive stump necrosis or ischemic non-viability, uncontrolled or progressive infection, and failure of local disease control after BKA.

Progressive stump necrosis or ischemic non-viability was defined as documented progression of tissue necrosis, wound deterioration, or loss of viable residual-limb tissue considered clinically attributable to inadequate perfusion. Uncontrolled or progressive infection was defined according to the treating team's documented assessment of persistent or worsening local or systemic signs of infection. Failure of local disease control was defined as continued deterioration of the stump or surrounding tissues such that a viable, closable, and potentially functional transtibial residual limb could no longer be maintained.

Because outcome decisions were made during routine clinical care, the treating team was not blinded to the patients’ clinical findings or laboratory results, including preoperative neutrophil-to-lymphocyte ratio and C-reactive protein values. Patients were categorized according to the presence or absence of progression to AKA for descriptive and comparative analyses. All patients included in the final analytic cohort underwent regular postoperative outpatient follow-up throughout the 3-month outcome-assessment period.

### 2.4 Data collection

Demographic, clinical, laboratory, operative, and postoperative data were obtained from the electronic medical records. The following variables were recorded: age, sex, body mass index (BMI), glycated hemoglobin (HbA1c), C-reactive protein (CRP), serum albumin, complete blood count parameters, Wagner grade, WIfI stage, amputation side, therapeutic antibiotic use before surgery, additional surgical procedures performed during follow-up, and original 12-Item Short Form Health Survey Version 1 (SF-12v1) scores.

Preoperative therapeutic antibiotic use was defined as systemic antimicrobial treatment initiated before the index operation for an active or suspected diabetic foot infection and was recorded separately from the standardized perioperative cefazolin prophylaxis administered to all patients.

Preoperative laboratory tests were obtained within 24 h before the index BKA. The neutrophil-to-lymphocyte ratio (NLR) was calculated from a single preoperative complete blood count by dividing the absolute neutrophil count by the absolute lymphocyte count. Average values from repeated measurements were not used. When more than one complete blood count was available within the preoperative period, the value closest to surgery was used.

For patients who progressed to AKA, operative notes and medical records were reviewed to determine the documented indication for proximal amputation. Intervening surgical procedures performed before conversion, including debridement and BKA stump revision, were also recorded.

### 2.5 Clinical assessment

Preoperative Wagner grades (5) were assigned by an orthopedic surgeon according to the Wagner classification system. All Wagner classifications were assigned by the same orthopedic surgeon.

Preoperative Wound, Ischemia, and foot Infection (WIfI) stages (6) were assigned by an experienced cardiovascular surgeon according to the Society for Vascular Surgery WIfI classification framework as part of routine clinical care. All WIfI evaluations were performed by the same cardiovascular surgeon. At the time of clinical assessment, the ischemia component was graded using the available objective perfusion measurements in accordance with the WIfI framework, together with clinical examination, wound characteristics, infection status, Doppler ultrasonography findings, and vascular specialist evaluation.

Preoperative vascular evaluation was performed in all patients before the index BKA and, when applicable, before subsequent revision or higher-level amputation procedures. Although the WIfI stage assignments were documented for all patients, the individual objective perfusion values underlying the ischemia component, including ankle–brachial index, toe–brachial index, toe pressure, or transcutaneous oxygen pressure, were not consistently retrievable from the retrospective medical records. Consequently, these measurements could not be analyzed as separate variables.

Interobserver reliability was not evaluated because Wagner and WIfI classifications were each assigned by a single experienced specialist.

Health-related quality of life was assessed using the original 12-Item Short Form Health Survey (SF-12v1) (11). Both the physical component score (PCS) and mental component score (MCS) were assessed before the index BKA and at postoperative week 3. SF-12v1 evaluations were performed by the same physical medicine and rehabilitation specialist and calculated according to standard scoring procedures, with higher scores indicating better health status. In patients who subsequently required amputation-level progression, the postoperative SF-12v1 assessment was performed approximately three weeks after the index BKA and before conversion to AKA. Because the standard SF-12v1 uses a four-week recall period, the postoperative week-3 assessment may have included approximately one week preceding the index BKA.

### 2.6 Surgical technique and perioperative and postoperative management

All BKA procedures were performed by a surgical team comprising two experienced orthopedic surgeons and two orthopedic surgery residents from the same institution, using a standardized transtibial amputation technique. The suitability of the transtibial amputation level was assessed preoperatively on the basis of the proximal extent of infection and tissue necrosis, the availability of viable soft tissue for stump coverage and closure, clinical perfusion findings, Doppler ultrasonography, and vascular surgery evaluation. A posterior flap technique was routinely used for stump formation, and soft-tissue coverage and stump closure were performed according to standard institutional surgical principles. Closed-suction drainage was routinely used as part of standard stump management. The surgical technique and postoperative stump care principles were standardized within the clinic, and no major surgeon-specific technical differences were documented.

Standardized perioperative antibiotic prophylaxis was administered according to the institutional protocol. A single preoperative dose of 2 g intravenous cefazolin was given before surgery, followed by intravenous cefazolin at a total daily dose of 4 g for three days postoperatively. In patients with persistent infection, progressive stump complications, or positive microbiological culture results, antibiotic therapy was modified or extended according to culture and antimicrobial susceptibility results and infectious disease consultation when available.

Postoperatively, patients were evaluated by a physical medicine and rehabilitation specialist and enrolled in a rehabilitation program. The program included stump care education, prevention of knee flexion contracture, bed mobility, transfer training, early mobilization as tolerated, strengthening exercises, and referral for prosthetic evaluation when clinically appropriate.

### 2.7 Statistical analysis

Statistical analyses were performed using IBM SPSS Statistics version 26.0 (IBM Corp., Armonk, NY, USA).

Continuous variables were expressed as mean ± standard deviation for normally distributed data and as median with interquartile range for non-normally distributed data. Categorical variables were presented as numbers and percentages. The normality of continuous variables was assessed using the Shapiro–Wilk test.

Comparisons between patients with and without progression of the amputation level were performed using the independent-samples *t*-test or Mann–Whitney *U* test for continuous variables, as appropriate. Categorical variables were compared using the Pearson chi-square test or Fisher's exact test, as appropriate.

To reduce the risk of model overfitting, a parsimonious multivariable logistic regression model was constructed using a limited number of clinically relevant covariates selected *a priori*. The dependent variable was progression from below-knee amputation (BKA) to above-knee amputation (AKA) within 3 months. The primary model included age, WIfI stage, Wagner grade, C-reactive protein (CRP), serum albumin, and neutrophil-to-lymphocyte ratio (NLR). These covariates were selected to represent age, local limb-threat severity, systemic inflammation, nutritional status, and the biomarker of primary interest.

Body mass index, sex, HbA1c, and preoperative therapeutic antibiotic use were not included in the final multivariable model to limit overfitting in view of the sample size and number of outcome events. These variables were not considered primary adjustment covariates because they were not regarded as direct markers of stump viability or local diabetic foot severity in the context of early amputation-level progression. In addition, baseline comparisons showed no statistically significant differences between patients with and without amputation-level progression in BMI, sex, HbA1c, or preoperative therapeutic antibiotic use.

CRP was modeled per 10 mg/L increase to improve the clinical interpretability of its odds ratio, whereas NLR was modeled per one-unit increase. WIfI stage 3 and Wagner grade 4 were used as the reference categories.

Because individual objective perfusion measurements, including ankle–brachial index, toe–brachial index, toe pressure, and transcutaneous oxygen pressure, were not consistently retrievable from the retrospective records, an additional sensitivity logistic regression model excluding WIfI stage was performed. This analysis was conducted to determine whether the association between preoperative NLR and amputation-level progression remained consistent when WIfI stage was omitted from the model.

To further evaluate model stability and the potential influence of the inverse CRP and Wagner grade coefficients, additional sensitivity logistic regression models were constructed by excluding CRP, excluding Wagner grade, and excluding both CRP and Wagner grade from the primary model. The adjusted odds ratio for NLR was compared across these model specifications. Multicollinearity among the covariates included in the primary model was assessed using variance inflation factors.

Overall model fit and apparent model performance were evaluated using the likelihood ratio test, Nagelkerke *R*^2^, the Hosmer–Lemeshow goodness-of-fit test, and the apparent area under the receiver operating characteristic curve. A calibration plot was generated to illustrate the apparent agreement between predicted probabilities and observed rates of amputation-level progression. Patients were grouped into deciles according to their predicted probabilities, and the observed progression rate in each decile was plotted against the corresponding mean predicted probability. Given the sample size, each decile contained approximately 10 patients; therefore, the calibration plot was considered a descriptive and potentially unstable within-sample assessment.

Because these model-performance measures were derived from the same dataset used to develop the model, they were regarded as apparent estimates. No bootstrap optimism correction, cross-validation, or external validation was performed. The Hosmer–Lemeshow test was used as a descriptive assessment of model fit. Given the limited sample size, a nonsignificant result was interpreted as indicating that lack of fit was not detected rather than as evidence of good calibration.

Spearman's rank correlation analysis was performed as an exploratory analysis to evaluate the relationships between preoperative NLR and selected demographic, metabolic, inflammatory, nutritional, and diabetic foot severity variables, including age, BMI, HbA1c, CRP, serum albumin, WIfI stage, and Wagner grade.

Receiver operating characteristic curve analysis was performed as an exploratory secondary analysis to evaluate the apparent discriminative ability of NLR alone for amputation-level progression. A cohort-specific cutoff was identified using the Youden index. Because the cutoff and its corresponding sensitivity and specificity were derived and evaluated in the same dataset, these estimates were considered exploratory and potentially optimistic and were not interpreted as a validated clinical decision threshold.

As an additional exploratory analysis, patients were classified into four groups according to combined preoperative NLR and serum albumin status. Elevated NLR was defined using the ROC-derived cutoff of 7.25, and hypoalbuminemia was defined as a serum albumin concentration <3.5 g/dL. Amputation-level progression rates were compared across the four groups using the Pearson chi-square test, and the effect size was calculated using Cramer's *V*. In addition, a logistic regression model including elevated NLR status, hypoalbuminemia status, and their interaction term was used to explore whether albumin status modified the association between elevated NLR and amputation-level progression.

Baseline comparisons, exploratory correlation analyses, ROC cutoff analysis, and subgroup analyses were not adjusted for multiple comparisons and should therefore be interpreted as exploratory.

There were no missing data for the variables included in the final analyses; therefore, no imputation procedure was performed. All statistical tests were two-tailed, and a *p* value <0.05 was considered statistically significant.

### 2.8 Ethical approval

The study was approved by the institutional ethics committee (Approval No: 3229; Date: 27 April 2021) and was conducted in accordance with the principles of the Declaration of Helsinki.

## 3 Results

### 3.1 Study population and baseline characteristics

A total of 105 patients who underwent below-knee amputation for diabetic foot complications were retrospectively included in the study. Preoperative laboratory parameters, including neutrophil and lymphocyte counts, were available for all patients, allowing calculation of the neutrophil-to-lymphocyte ratio (NLR). Demographic data, metabolic parameters, inflammatory markers, and disease severity scores were extracted from electronic medical records. There were no missing data for age, sex, BMI, HbA1c, CRP, albumin, neutrophil count, lymphocyte count, NLR, Wagner grade, WIfI stage, amputation side, therapeutic antibiotic use before surgery, postoperative antibiotic use, or SF-12v1 scores among the 105 patients included in the final analysis.

During the 3-month follow-up period, 52 patients (49.5%) maintained the initial BKA level, whereas 53 patients (50.5%) experienced progression from BKA to AKA. Outcomes were subsequently compared according to the presence or absence of amputation-level progression.

### 3.2 Indications for amputation**-**level progression and intervening procedures

Among the 53 patients who progressed from BKA to AKA within 3 months, the documented indications for proximal amputation were persistent or progressive infection alone in 7 patients (13.2%), stump necrosis or ischemic non-viability alone in 4 patients (7.5%), and combined infection and necrosis in 42 patients (79.2%).

Before conversion to AKA, 20 patients (37.7%) underwent at least one intervening surgical procedure. Debridement was performed in 19 patients (35.8%), and BKA stump revision was performed in 3 patients (5.7%). Two of these patients underwent both debridement and stump revision. The remaining 33 patients (62.3%) proceeded directly to AKA without an intervening surgical procedure.

### 3.3 Baseline demographic, clinical, and laboratory characteristics

The baseline demographic, clinical, laboratory, and health-related quality-of-life characteristics of the study population are summarized in Table 1. Patients with and without amputation-level progression were comparable with respect to age, BMI, sex distribution, and side of amputation.

**Table 1:** Demographic, clinical, laboratory, treatment, and health-related quality-of-life characteristics of the study population.

Variable	BKA maintained (*n* = 52)	BKA → AKA progression (*n* = 53)	*p* value
Age (years, mean ± SD)	61.9 ± 10.7	64.7 ± 10.4	0.180
BMI (kg/m^2^, mean ± SD)	29.3 ± 5.3	29.9 ± 5.7	0.580
Sex, *n* (%)			0.081
Male	37 (71.2%)	29 (54.7%)	
Female	15 (28.8%)	24 (45.3%)	
Side, *n* (%)			0.479
Right	32 (61.5%)	29 (54.7%)	
Left	20 (38.5%)	24 (45.3%)	
Wagner grade, *n* (%)			0.206
4	24 (46.2%)	31 (58.5%)	
5	28 (53.8%)	22 (41.5%)	
WIfI stage, *n* (%)			0.383
3	26 (50.0%)	22 (41.5%)	
4	26 (50.0%)	31 (58.5%)	
CRP [mg/L, median (IQR)]	109 (62.0–191.9)	126 (39.0–178.0)	0.656
Neutrophil count ( × 10^9^/L), median [IQR]	10.52 [5.79–14.04]	12.30 [9.21–17.80]	**0**.**006**
Lymphocyte count ( × 10^9^/L), median [IQR]	1.79 [1.35–2.14]	1.40 [1.13–1.84]	**0**.**006**
NLR, median [IQR]	5.47 (3.24–9.04)	10.42 (5.68–13.03)	**<0**.**001**
HbA1c (%, mean ± SD)	8.99 ± 2.19	8.87 ± 2.82	0.813
Albumin (g/dL, mean ± SD)	3.01 ± 0.83	2.97 ± 0.97	0.805
Preoperative therapeutic antibiotic use (yes), *n* (%)	32 (61.5%)	36 (67.9%)	0.493
Postoperative antibiotics (yes), *n* (%)	52 (100%)	53 (100%)	—
Preoperative SF-12v1 PCS (mean ± SD)	35.43 ± 4.66	36.29 ± 4.77	0.350
Preoperative SF-12v1 MCS (mean ± SD)	52.89 ± 5.05	52.12 ± 6.46	0.496
Postoperative SF-12v1 PCS (mean ± SD)	31.49 ± 4.14	32.60 ± 4.08	0.171
Postoperative SF-12v1 MCS (mean ± SD)	47.08 ± 5.08	42.79 ± 6.75	**<0**.**001**

Values are presented as mean ± standard deviation for normally distributed variables, median (interquartile range) for non-normally distributed variables, and number (%) for categorical variables. Continuous variables were compared using the independent-samples *t*-test or Mann–Whitney *U* test, as appropriate. Categorical variables were compared using the Pearson chi-square test or Fisher's exact test, as appropriate. CRP, neutrophil count, lymphocyte count, and NLR were non-normally distributed and are therefore presented as median (interquartile range).

Bold *p* values indicate statistical significance (*p* < 0.05).

BKA, below-knee amputation; AKA, above-knee amputation; BMI, body mass index; WIfI, wound, ischemia, and foot infection; CRP, C-reactive protein; NLR, neutrophil-to-lymphocyte ratio; HbA1c, glycated hemoglobin; SF-12v1, 12-Item Short Form Health Survey; PCS, physical component score; MCS, mental component score.

The mean age was 61.9 ± 10.7 years in patients without amputation-level progression and 64.7 ± 10.4 years in patients with amputation-level progression, with no statistically significant difference according to progression status (*p* = 0.180). Similarly, BMI did not differ significantly according to progression status (29.3 ± 5.3 kg/m^2^ vs. 29.9 ± 5.7 kg/m^2^, *p* = 0.580).

Sex distribution was also comparable according to progression status. Male patients accounted for 71.2% of patients without amputation-level progression and 54.7% of patients with amputation-level progression, whereas female patients represented 28.8% and 45.3%, respectively, with no statistically significant difference (*p* = 0.081). Likewise, no statistically significant difference was observed regarding the side of amputation according to progression status (*p* = 0.479).

### 3.4 Disease severity and conventional laboratory findings

Disease severity parameters and conventional laboratory findings are presented in Table 1. Wagner grade distribution did not differ significantly according to amputation-level progression status. Among patients without amputation-level progression, 24 patients (46.2%) had Wagner grade 4 and 28 patients (53.8%) had Wagner grade 5 disease. Among patients with amputation-level progression, 31 patients (58.5%) had Wagner grade 4 and 22 patients (41.5%) had Wagner grade 5 disease (*p* = 0.206).

Similarly, WIfI stage distribution did not differ significantly according to amputation-level progression status. WIfI stage 3 was observed in 26 patients (50.0%) without amputation-level progression and 22 patients (41.5%) with amputation-level progression, while WIfI stage 4 was present in 26 patients (50.0%) and 31 patients (58.5%), respectively (*p* = 0.383).

CRP levels did not differ significantly according to amputation-level progression status [109 (62.0–191.9) vs. 126 (39.0–178.0) mg/L, *p* = 0.656]. Similarly, HbA1c values were comparable between patients without and with amputation-level progression (8.99 ± 2.19% vs. 8.87 ± 2.82%, *p* = 0.813). Serum albumin levels were also similar between patients without and with amputation-level progression (3.01 ± 0.83 g/dL vs. 2.97 ± 0.97 g/dL, *p* = 0.805).

The proportion of patients receiving preoperative therapeutic antibiotics did not differ significantly according to amputation-level progression status (61.5% vs. 67.9%, *p* = 0.493). Postoperative antibiotic therapy was administered to all patients.

Patients with amputation-level progression had higher preoperative neutrophil counts [12.30 (9.21–17.80) vs. 10.52 (5.79–14.04) × 10^9^/L, *p* = 0.006] and lower lymphocyte counts [1.40 (1.13–1.84) vs. 1.79 (1.35–2.14) × 10^9^/L, *p* = 0.006] than patients without progression.

### 3.5 Neutrophil-to-lymphocyte ratio findings

**Figure 1:**
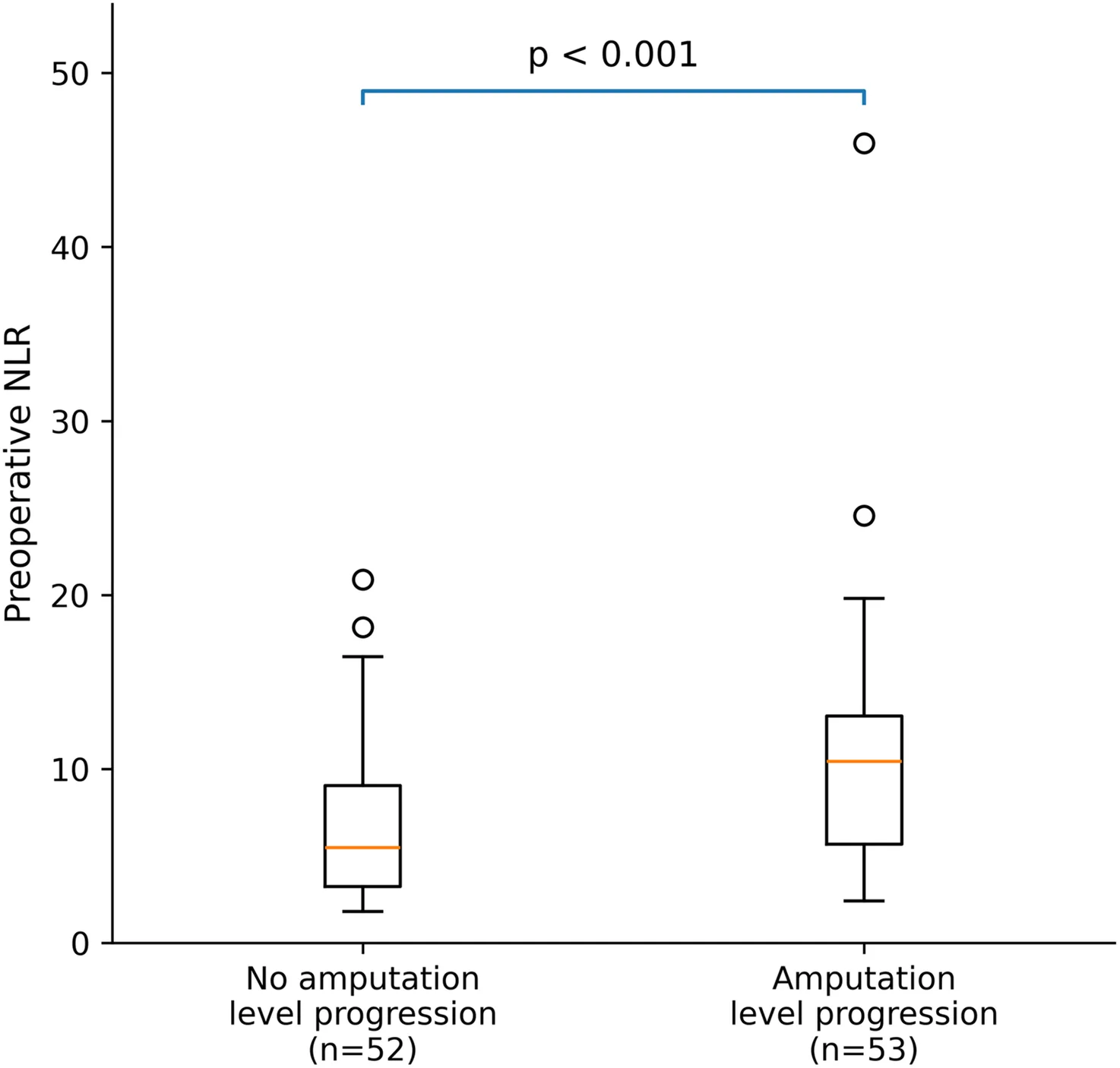
Distribution of preoperative neutrophil-to-lymphocyte ratio according to amputation-level progression. Preoperative NLR values were significantly higher in patients who experienced progression from below-knee to above-knee amputation than in those who maintained the initial below-knee amputation level (*p* < 0.001). Boxes represent the interquartile range, horizontal lines indicate medians, whiskers indicate non-outlier ranges, and circles indicate outliers. The *p*-value was calculated using the Mann–Whitney *U* test. NLR, neutrophil-to-lymphocyte ratio.

NLR differed significantly according to amputation level-progression status. The median NLR was 5.47 (3.24–9.04) in patients without amputation-level progression and 10.42 (5.68–13.03) in patients with amputation-level progression (*p* < 0.001) (Figure 1).

In exploratory correlation analysis, preoperative NLR showed a moderate positive correlation with CRP (Spearman's rho = 0.528, *p* < 0.001). No statistically significant correlations were observed between NLR and age, BMI, HbA1c, serum albumin, WIfI stage, or Wagner grade (Table 2).

**Table 2:** Exploratory correlations between preoperative NLR and clinical/laboratory variables.

Variable correlated with NLR	Spearman's rho	*p* value
Age	0.088	0.370
BMI	0.154	0.117
HbA1c	−0.065	0.508
CRP	0.528	**<0** **.** **001**
Serum albumin	−0.028	0.777
WIfI stage	−0.080	0.415
Wagner grade	−0.120	0.223

Spearman's correlation analysis was performed to evaluate the relationships between preoperative NLR and selected demographic, metabolic, inflammatory, nutritional, and diabetic foot severity variables. A two-sided *p*-value < 0.05 was considered statistically significant.

Bold *p* values indicate statistical significance.

NLR, neutrophil-to-lymphocyte ratio; BMI, body mass index; HbA1c, glycated hemoglobin; CRP, C-reactive protein; WIfI, wound, ischemia, and foot infection.

### 3.6 Exploratory ROC analysis

**Figure 2:**
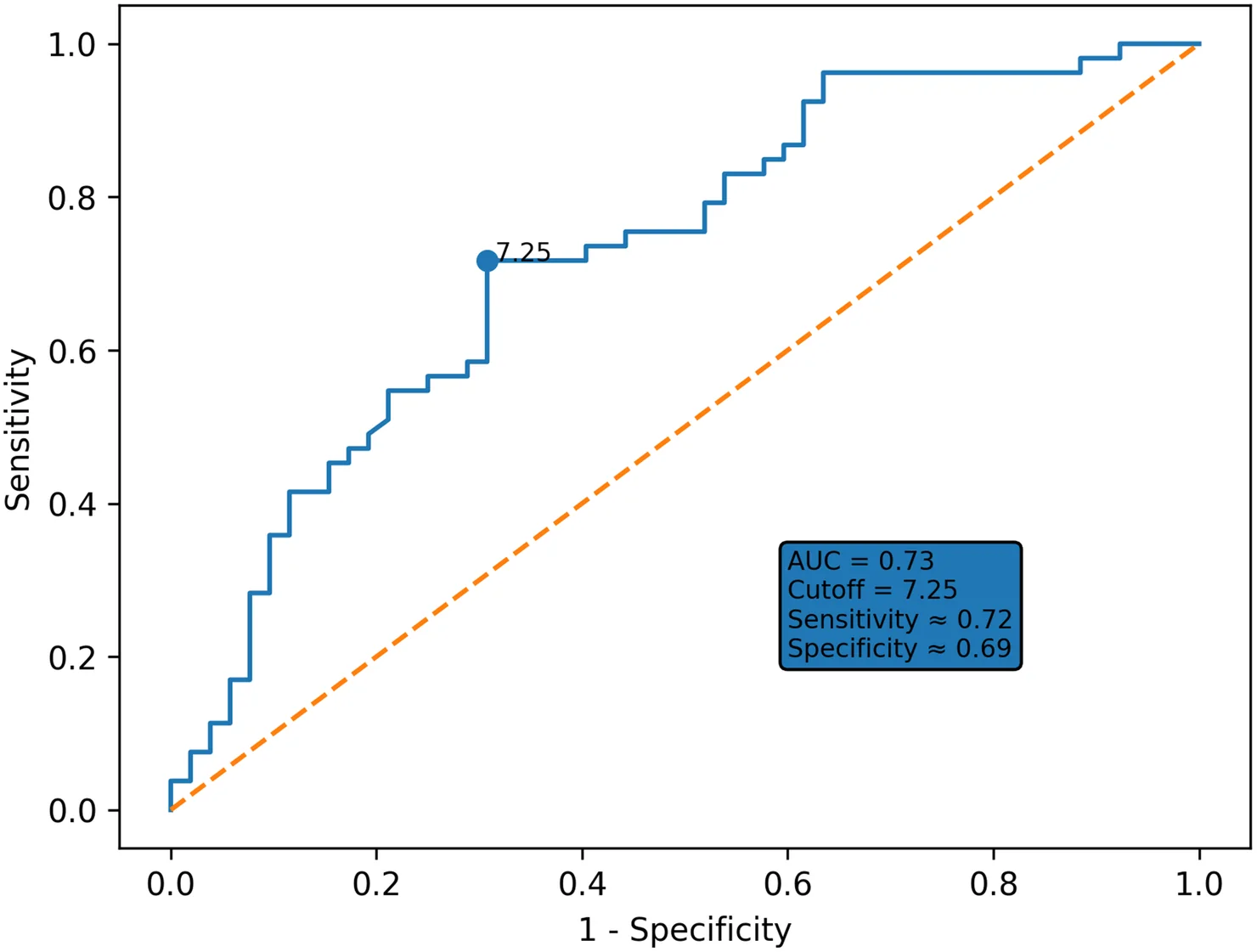
Exploratory receiver operating characteristic curve of preoperative neutrophil-to-lymphocyte ratio for amputation-level progression. The area under the curve was 0.73, indicating moderate apparent discriminative ability. The Youden index identified a cohort-specific NLR cutoff of 7.25, corresponding to a sensitivity of 71.7% and a specificity of 69.2%. The cutoff point is indicated on the ROC curve. This Youden-derived cutoff represents an exploratory within-sample estimate and should not be interpreted as a validated clinical decision threshold. NLR, neutrophil-to-lymphocyte ratio; ROC, receiver operating characteristic.

Receiver operating characteristic (ROC) curve analysis was performed to explore the apparent discriminative ability of preoperative NLR for amputation-level progression (Figure 2).

The area under the curve (AUC) was 0.73, indicating moderate apparent discrimination between patients with and without amputation-level progression.

Using Youden's index, a cohort-specific NLR cutoff of 7.25 was identified. At this cutoff, sensitivity was 71.7% and specificity was 69.2%. Because the cutoff and performance estimates were derived from the same cohort, these findings should be considered exploratory and were not interpreted as a validated clinical decision threshold.

### 3.7 Multivariable logistic regression analysis

**Figure 3:**
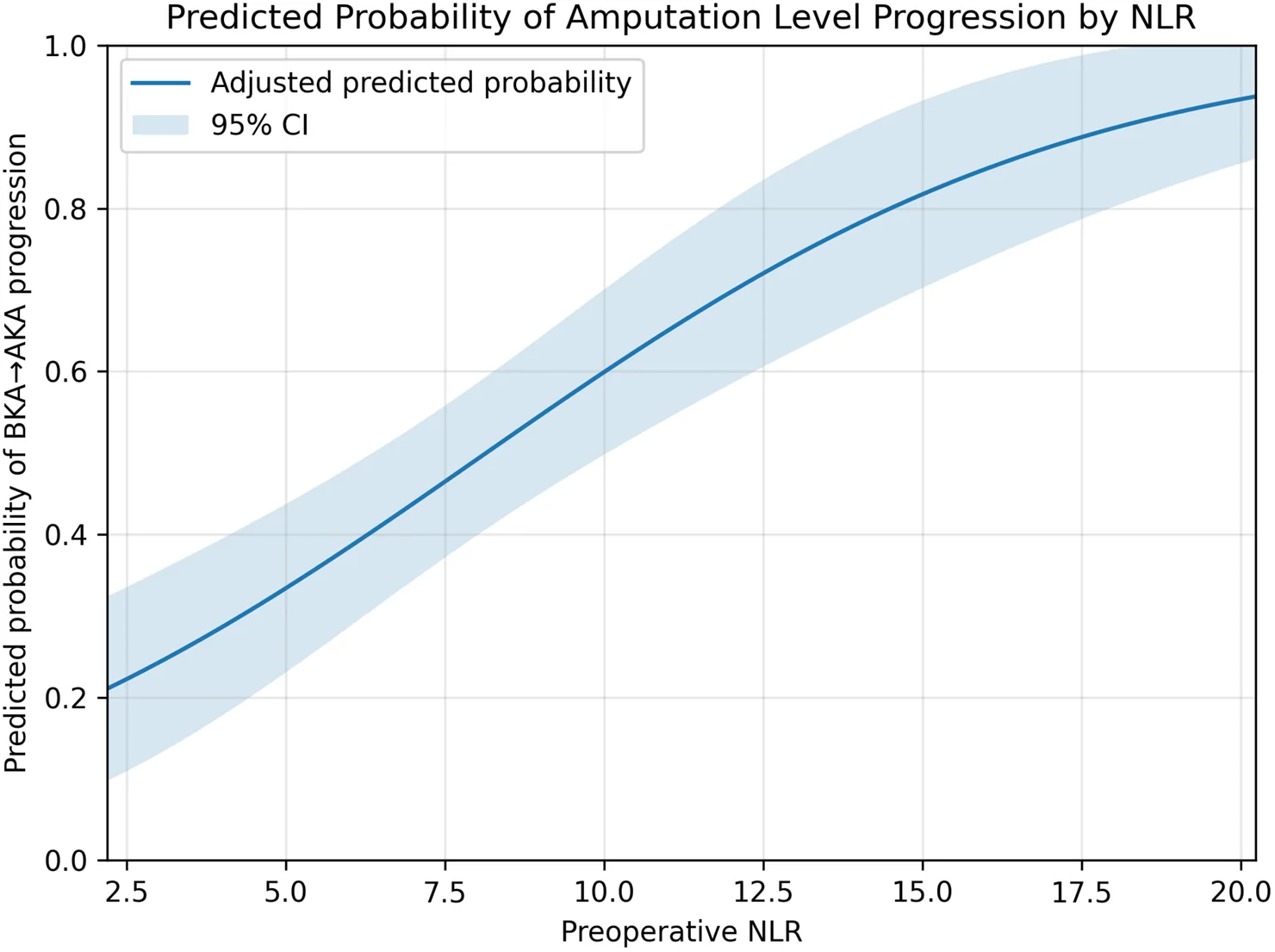
Adjusted predicted probability of amputation-level progression according to preoperative NLR. The figure shows the adjusted predicted probability of progression from below-knee to above-knee amputation within 3 months across increasing preoperative NLR values. Predicted probabilities were derived from the multivariable logistic regression model including age, WIfI stage, Wagner grade, CRP, serum albumin, and NLR. The shaded area represents the 95% confidence interval. Predicted probabilities were used to illustrate the adjusted association between preoperative NLR and amputation-level progression and should not be interpreted as externally validated individual risk estimates. NLR, neutrophil-to-lymphocyte ratio; WIfI, wound, ischemia, and foot Infection; CRP, C-reactive protein.

In the parsimonious multivariable logistic regression model including age, WIfI stage, Wagner grade, CRP, serum albumin, and NLR, preoperative NLR remained significantly associated with amputation-level progression. Each one-unit increase in preoperative NLR was associated with a 29% increase in the odds of progression from BKA to AKA within 3 months after adjustment for age, WIfI stage, Wagner grade, CRP, and serum albumin (adjusted OR: 1.29, 95% CI: 1.14–1.47, *p* < 0.001) (Table 3). The adjusted predicted probability plot showed a progressive increase in the estimated probability of amputation-level progression with increasing preoperative NLR values (Figure 3). This within-sample plot was presented for illustrative purposes and should not be interpreted as a validated prediction tool.

**Table 3:** Parsimonious multivariable logistic regression model for factors associated with amputation-level progression from below-knee to above-knee amputation.

Variable	*β*	SE	Adjusted OR	95% CI	*p* value
Age, per year increase	0.022	0.022	1.02	0.98–1.07	0.318
WIfI stage 4 vs. 3	0.912	0.533	2.49	0.88–7.08	0.087
Wagner grade 5 vs. 4	−0.940	0.533	0.39	0.14–1.11	0.078
CRP, per 10 mg/L increase	−0.087	0.031	0.92	0.86–0.97	**0**.**006**
Serum albumin, per 1 g/dL increase	0.039	0.263	1.04	0.62–1.74	0.883
NLR, per unit increase	0.257	0.065	1.29	1.14–1.47	<0.001

BKA, below-knee amputation; AKA, above-knee amputation; OR, odds ratio; CI, confidence interval; SE, standard error; WIfI, wound, ischemia, and foot Infection; CRP, C-reactive protein; NLR, neutrophil-to-lymphocyte ratio; AUC, area under the receiver operating characteristic curve.

Multivariable logistic regression was performed using a parsimonious model to reduce the risk of overfitting. Variables were selected a priori based on clinical relevance and included age, WIfI stage, Wagner grade, CRP, serum albumin, and NLR. CRP was modeled per 10 mg/L increase, whereas NLR was modeled per unit increase. WIfI stage 3 and Wagner grade 4 were used as the reference categories. The dependent variable was amputation-level progression from BKA to AKA within 3 months. WIfI stage was assigned during routine clinical care by an experienced cardiovascular surgeon according to the society for vascular surgery WIfI classification framework; individual objective perfusion values were not consistently retrievable from the retrospective records.

Model summary: Nagelkerke *R*^2^ = 0.318; Hosmer–Lemeshow goodness-of-fit test, *χ*^2^ = 13.32, *df* = 8, *p* = 0.101; overall likelihood ratio test, *χ*^2^ = 28.66, *df* = 6, *p* < 0.001; apparent model AUC = 0.792.

Bold *p* values indicate statistical significance (*p* < 0.05).

Because individual objective perfusion values underlying WIfI assessment were not consistently retrievable from the retrospective records, an additional sensitivity analysis excluding WIfI stage was performed (Table 4). In this sensitivity model including age, Wagner grade, CRP, serum albumin, and NLR, preoperative NLR remained significantly associated with amputation-level progression, with an effect estimate very similar to that of the primary model (adjusted OR: 1.29, 95% CI: 1.13–1.46, *p* < 0.001). The apparent AUC was also similar after exclusion of WIfI stage compared with the primary model (0.787 vs. 0.792).

**Table 4:** Sensitivity analyses of the association between preoperative NLR and amputation-level progression.

Model	Covariates included	Adjusted OR for NLR	95% CI	*p* value	Apparent AUC
Primary model	Age, WIfI stage, Wagner grade, CRP, serum albumin, NLR	1.29	1.14–1.47	**<0** **.** **001**	0.792
Model excluding WIfI stage	Age, Wagner grade, CRP, serum albumin, NLR	1.29	1.13–1.46	**<0** **.** **001**	0.787
Model excluding CRP	Age, WIfI stage, Wagner grade, serum albumin, NLR	1.17	1.07–1.29	**0** **.** **001**	0.745
Model excluding Wagner grade	Age, WIfI stage, CRP, serum albumin, NLR	1.29	1.14–1.46	**<0** **.** **001**	0.776
Model excluding CRP and Wagner grade	Age, WIfI stage, serum albumin, NLR	1.17	1.07–1.29	**0** **.** **001**	0.734

NLR, neutrophil-to-lymphocyte ratio; WIfI, wound, ischemia, and foot Infection; CRP, C-reactive protein; OR, odds ratio; CI, confidence interval; AUC, area under the receiver operating characteristic curve. Adjusted ORs for NLR are reported per one-unit increase. CRP was entered into the models per 10 mg/L increase. Wagner grade was entered as grade 5 vs. grade 4, and WIfI stage was entered as stage 4 vs. stage 3. Apparent AUC represents model discrimination estimated in the same dataset used to fit the models, without internal or external validation.

Bold *p* values indicate statistical significance (*p* < 0.05).

To further assess model stability, variance inflation factors and additional sensitivity models were evaluated. Variance inflation factors in the primary model were low, with a maximum VIF of 1.38, indicating no substantial multicollinearity among the model covariates. In sensitivity analyses, preoperative NLR remained significantly associated with amputation-level progression after exclusion of CRP, exclusion of Wagner grade, and exclusion of both CRP and Wagner grade (Table 4). The NLR effect estimate was nearly unchanged after exclusion of Wagner grade, whereas it was attenuated after exclusion of CRP but remained statistically significant. These findings indicated that the association between preoperative NLR and amputation-level progression was directionally consistent across alternative model specifications.

CRP was also statistically significant in the adjusted model (adjusted OR: 0.92 per 10 mg/L increase, 95% CI: 0.86–0.97, *p* = 0.006). However, because the direction of the adjusted association was inverse and CRP did not differ significantly according to progression status in the unadjusted baseline comparison, this finding was interpreted cautiously and was not considered evidence of a protective effect. WIfI stage 4 showed numerically higher odds of amputation-level progression than WIfI stage 3, but this association did not reach statistical significance (adjusted OR: 2.49, 95% CI: 0.88–7.08, *p* = 0.087). Wagner grade 5 showed numerically lower odds of progression than Wagner grade 4, although this association was not statistically significant (adjusted OR: 0.39, 95% CI: 0.14–1.11, *p* = 0.078). Age and serum albumin were not significantly associated with amputation-level progression in the adjusted model.

**Figure 4:**
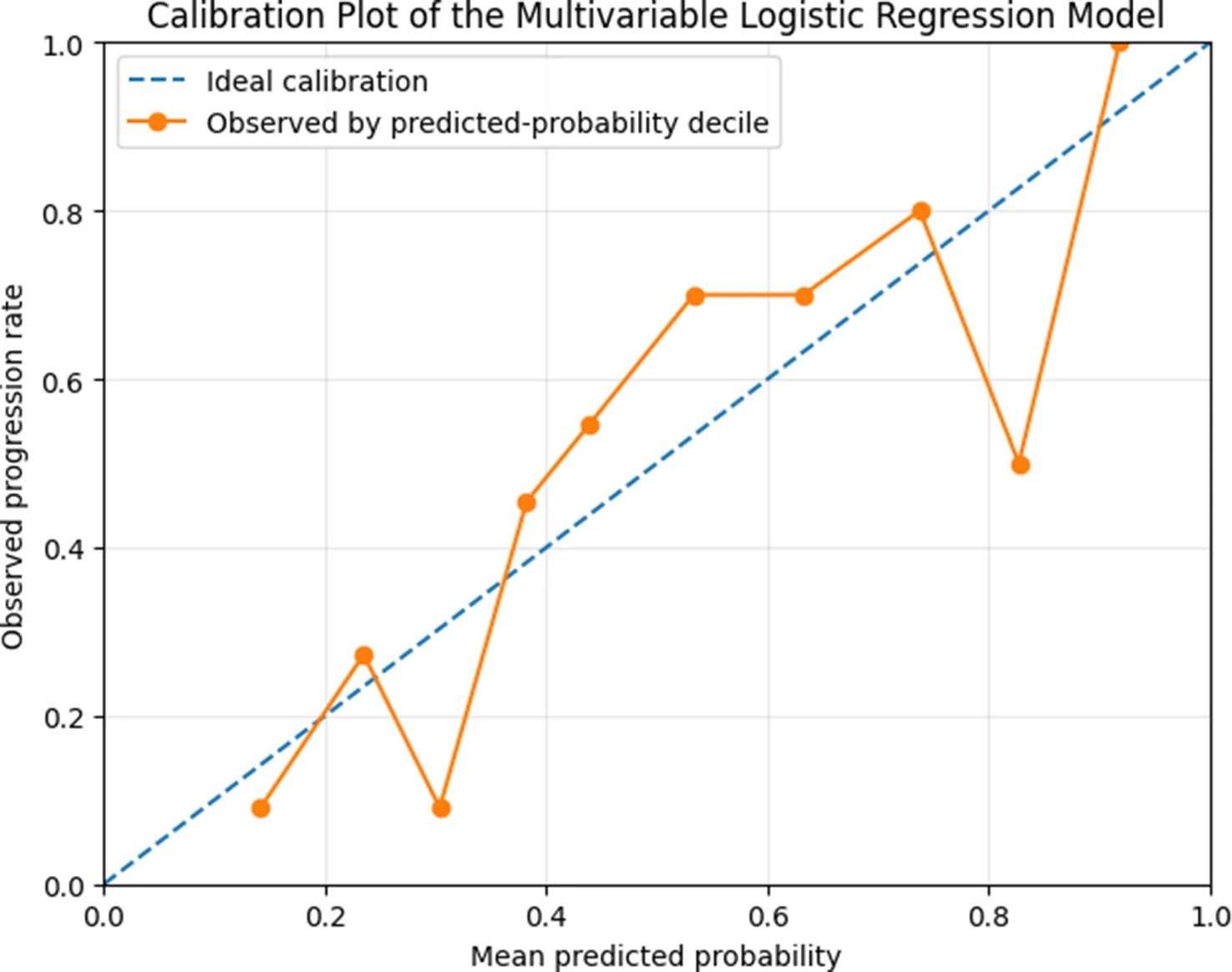
Apparent calibration plot of the multivariable logistic regression model. Patients were grouped into deciles according to predicted probability, and observed amputation-level progression rates were plotted against the mean predicted probability in each decile. The dashed diagonal line represents ideal calibration. The Hosmer–Lemeshow test did not detect a statistically significant lack of fit (*χ*^2^ = 13.32, *df* = 8, *p* = 0.101). However, the plot should be interpreted cautiously because it was derived from the same dataset used to fit the model and was based on a limited number of patients within each decile.

The Hosmer–Lemeshow goodness-of-fit test did not detect a statistically significant lack of fit (*χ*^2^ = 13.32, *df* = 8, *p* = 0.101). However, given the limited sample size, this result should not be interpreted as definitive evidence of good calibration. The overall likelihood ratio test was statistically significant (*χ*^2^ = 28.66, *df* = 6, *p* < 0.001), indicating that the model provided a better fit than the intercept-only model. Nagelkerke *R*^2^ was 0.318, and the apparent model AUC was 0.792. The apparent decile-based calibration plot is presented in Figure 4. Given the limited sample size and the small number of patients within each decile, the plot was considered descriptive and potentially unstable and should not be interpreted as evidence of validated calibration.

### 3.8 Combined NLR and albumin analysis

To further explore the combined association of systemic inflammation and nutritional status with amputation-level progression, patients were stratified according to preoperative NLR and serum albumin levels.

**Figure 5:**
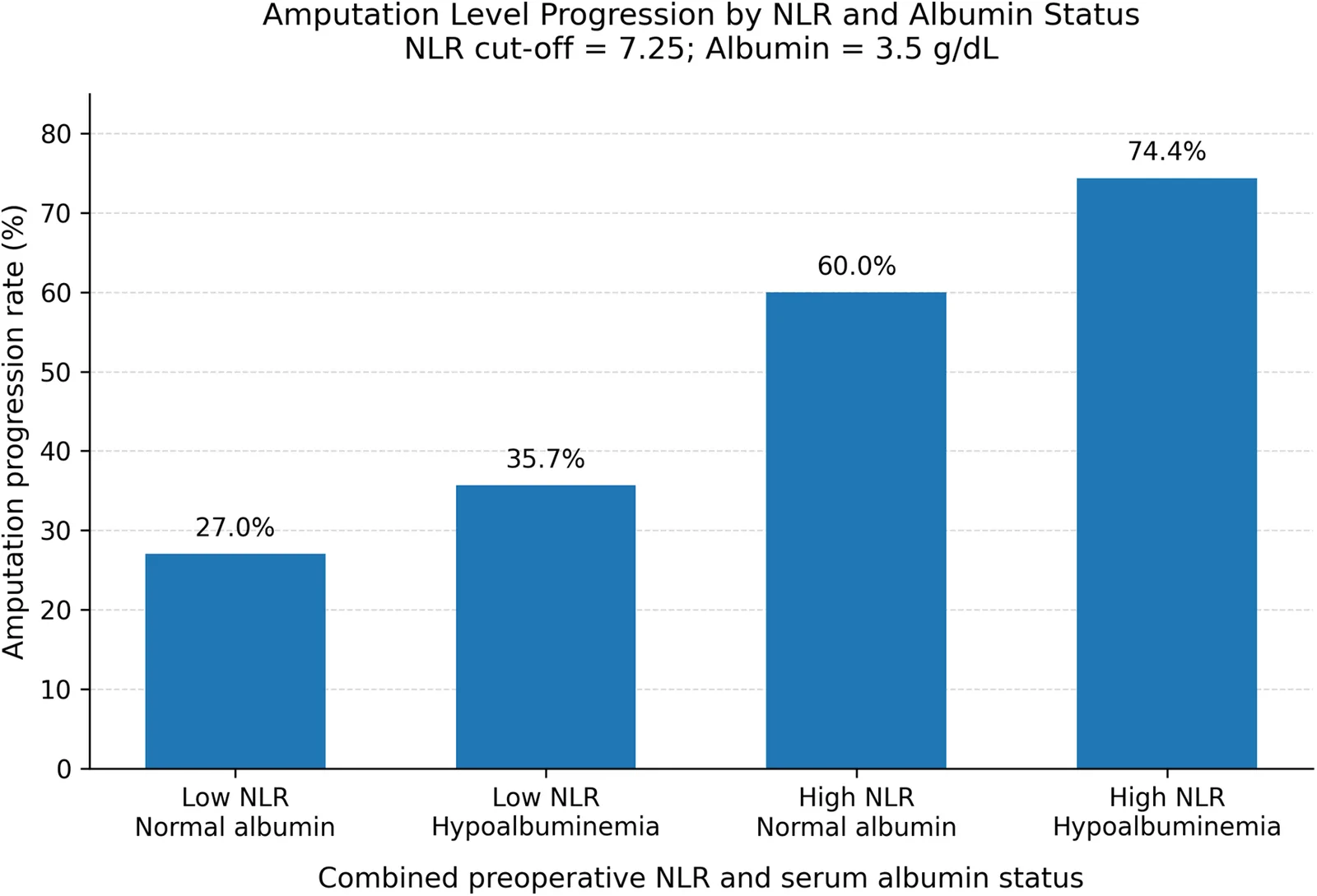
Amputation-level progression according to combined neutrophil-to-lymphocyte ratio and serum albumin status. Patients were stratified into four groups according to preoperative NLR and serum albumin levels. High NLR was defined as NLR ≥ 7.25 based on the exploratory ROC analysis, and hypoalbuminemia was defined as a serum albumin concentration <3.5 g/dL. Bars represent the proportion of patients who progressed from below-knee to above-knee amputation within 3 months, and values above the bars indicate progression rates. Progression rates differed significantly across the four groups according to the Pearson chi-square test (*χ*^2^ = 18.80, *df* = 3, *p* < 0.001; Cramer's *V* = 0.42). NLR, neutrophil-to-lymphocyte ratio; ROC, receiver operating characteristic.

Patients were classified into four groups according to preoperative NLR and serum albumin status, with high NLR defined as ≥7.25 and hypoalbuminemia defined as serum albumin <3.5 g/dL. Amputation-level progression occurred in 10 of 37 patients (27.0%) with low NLR and normal albumin, 5 of 14 patients (35.7%) with low NLR and hypoalbuminemia, 9 of 15 patients (60.0%) with high NLR and normal albumin, and 29 of 39 patients (74.4%) with high NLR and hypoalbuminemia. The highest progression rate was observed in patients with both high NLR and hypoalbuminemia, whereas the lowest rate was observed in patients with low NLR and normal albumin. Progression rates differed significantly across the four groups (*χ*^2^ = 18.80, *df* = 3, *p* < 0.001; Cramer's *V* = 0.42) (Figure 5). In the formal logistic regression analysis, the NLR × hypoalbuminemia interaction term was not statistically significant (interaction OR: 2.90, 95% CI: 0.47–17.86, *p* = 0.251), indicating that the observed four-group gradient did not provide evidence that hypoalbuminemia modified the association between elevated NLR and amputation-level progression.

### 3.9 SF-12v1 outcomes

Health-related quality of life was evaluated using the 12-Item Short Form Health Survey (SF-12v1). The preoperative SF-12v1 assessment was performed before the index BKA, and the postoperative assessment was performed approximately three weeks after surgery. In patients who subsequently underwent amputation-level progression, the postoperative assessment was completed before conversion to AKA.

Preoperative and postoperative physical component scores (PCS) and mental component scores (MCS) are summarized in Table 1. No statistically significant differences were observed according to progression status in preoperative PCS (35.43 ± 4.66 vs. 36.29 ± 4.77, *p* = 0.350) or preoperative MCS (52.89 ± 5.05 vs. 52.12 ± 6.46, *p* = 0.496).

Postoperative MCS was significantly lower in patients with amputation-level progression than in those who maintained the initial BKA level (42.79 ± 6.75 vs. 47.08 ± 5.08, *p* < 0.001). Postoperative PCS did not differ significantly according to progression status (32.60 ± 4.08 vs. 31.49 ± 4.14, *p* = 0.171).

## 4 Discussion

### 4.1 Main findings

This retrospective study evaluated the association between preoperative neutrophil-to-lymphocyte ratio (NLR) and early progression from below-knee amputation (BKA) to above-knee amputation (AKA) in patients with diabetic foot disease. Although the relationship between elevated NLR and adverse diabetic foot outcomes has been previously described (9, 10, 12, 13), the present study specifically focused on amputation-level progression after an initial BKA rather than on the occurrence of amputation alone. This distinction is clinically relevant because progression from BKA to AKA represents not only a more proximal final amputation level but also failure to maintain the initial amputation level, exposing patients to an additional major surgical procedure, delayed rehabilitation, prolonged recovery, and greater cumulative physical and psychological burden.

The early progression rate observed in this cohort was relatively high, with 53 of 105 patients (50.5%) undergoing conversion from BKA to AKA within 3 months. This rate should be interpreted in the context of the highly selected and clinically severe study population. All patients had already required major transtibial amputation and had advanced diabetic foot disease, with Wagner grades 4–5 and WIfI stages 3–4. In addition, combined infection and stump necrosis were documented in 42 of the 53 patients (79.2%) who progressed to AKA, indicating that the high progression rate occurred in a population with substantial infectious burden and compromised residual-limb tissue viability. The tertiary referral setting may also have contributed to the inclusion of a disproportionate number of patients with complex, advanced, or treatment-refractory disease. Furthermore, the exclusion of patients with insufficient 3-month follow-up and one patient who died before outcome ascertainment may have introduced selection bias and affected the observed progression rate. Death before outcome ascertainment also represents a potential competing event that could not be formally evaluated in this cohort. Accordingly, the progression rate observed in this study may not be directly comparable with conversion or revision rates reported in broader and less severely affected diabetic amputation populations.

In this cohort, available baseline indicators of local disease severity, metabolic status, systemic inflammation, nutritional status, and perioperative management, including WIfI stage, Wagner grade, HbA1c, CRP, serum albumin, and preoperative therapeutic antibiotic use, were broadly comparable according to amputation-level progression status. In the parsimonious multivariable logistic regression model adjusted for age, WIfI stage, Wagner grade, CRP, serum albumin, and NLR, preoperative NLR remained significantly associated with early amputation-level progression. Each one-unit increase in preoperative NLR was associated with 29% higher odds of progression from BKA to AKA within 3 months.

The inverse adjusted associations observed for CRP and Wagner grade should be interpreted cautiously. Although CRP was statistically significant in the adjusted model, its inverse direction was not clinically intuitive, and CRP did not differ significantly according to progression status in the unadjusted comparison. This finding may reflect shared inflammatory information captured by CRP and NLR, the effects of covariate adjustment, residual confounding, or model instability in a relatively small cohort. Similarly, Wagner grade 5 showed numerically lower odds of progression than Wagner grade 4. One possible explanation is that patients with Wagner grade 5 disease may have undergone a more extensive initial transtibial resection because of greater tissue loss or gangrene, thereby reducing the likelihood of subsequent proximal revision. Alternatively, this finding may reflect the limited variation in disease severity because the cohort included only patients with Wagner grades 4–5 and should not be interpreted as evidence that Wagner grade 5 is protective.

The documented indications for proximal amputation suggest that early amputation-level progression was multifactorial. In most patients who progressed to AKA, infection and stump necrosis or ischemic non-viability coexisted, whereas isolated infection or isolated necrosis was less common. This supports the concept that progression after the initial BKA may reflect the combined effects of persistent infection, impaired tissue viability, and local wound complications.

Patients with amputation-level progression had both higher preoperative neutrophil counts and lower lymphocyte counts, indicating that the observed difference in NLR was attributable to changes in both components of the ratio. Exploratory correlation analysis showed that NLR was moderately correlated with CRP, supporting its relationship with systemic inflammatory activity. In contrast, NLR was not significantly correlated with WIfI stage or Wagner grade, suggesting that it may reflect a systemic inflammatory component not fully represented by local diabetic foot severity classifications. Overall, these findings suggest that preoperative NLR may provide adjunctive information regarding early amputation-level progression, although its clinical utility requires confirmation in larger, externally validated cohorts.

### 4.2 Exploratory discriminative performance of NLR and comparison with previous studies

In the exploratory receiver operating characteristic (ROC) analysis, preoperative NLR showed moderate apparent discrimination between patients who maintained the initial below-knee amputation level and those who progressed to above-knee amputation within 3 months, with an area under the curve (AUC) of 0.73. The Youden index identified a cohort-specific NLR cutoff of 7.25, corresponding to a sensitivity of 71.7% and a specificity of 69.2%.

These findings suggest that NLR may provide supplementary information when considered alongside established clinical and laboratory parameters. However, the observed discrimination was moderate, and NLR should not be interpreted as a standalone prediction tool or clinical decision threshold. Because the cutoff and its corresponding sensitivity and specificity were derived and evaluated in the same cohort, these estimates are susceptible to optimism. The cutoff of 7.25 should therefore be regarded as exploratory and hypothesis-generating and requires validation in larger, independent cohorts before clinical application. Similarly, the adjusted probability estimates were used to illustrate the adjusted association between NLR and amputation-level progression rather than to provide individualized risk predictions.

Previous studies have also reported associations between elevated NLR and adverse outcomes in diabetic foot disease, including impaired wound healing and major amputation. Zhao et al. identified a preoperative NLR cutoff of 4.25 for predicting wound-healing time in patients with Wagner grade 3–4 diabetic foot ulcers undergoing tibial cortex transverse transport surgery (14). Lower thresholds have also been reported in studies evaluating diabetic wound healing (15), whereas higher thresholds have been reported in cohorts with more advanced disease or amputation-related outcomes. Afshar et al. reported that an NLR value greater than 6.08 was associated with an increased risk of amputation in patients with diabetic foot ulcers (16). Higher NLR thresholds have also been reported in some advanced diabetic foot cohorts, although estimates vary considerably across study populations (17).

Variation in reported NLR thresholds is likely attributable to differences in study populations, infection severity, vascular status, ulcer characteristics, treatment pathways, and outcome definitions. The relatively higher cutoff observed in the present study may reflect the inclusion of patients who had already undergone BKA, representing a population with advanced limb-threatening disease and a greater baseline inflammatory burden. Accordingly, the cutoff of 7.25 should be interpreted as specific to this cohort rather than as a universally applicable threshold.

NLR and CRP may also be affected by concurrent infection, systemic inflammation, and comorbid conditions. These biomarkers should therefore be interpreted in conjunction with vascular status, infection severity, metabolic control, nutritional status, and overall clinical severity.

### 4.3 Combined inflammatory and nutritional findings

In the exploratory subgroup analysis, patients with both elevated NLR and hypoalbuminemia had the highest observed rate of amputation-level progression. However, the formal NLR × hypoalbuminemia interaction was not statistically significant (interaction OR: 2.90, 95% CI: 0.47–17.86, *p* = 0.251), providing no evidence that albumin status modified the association between elevated NLR and amputation-level progression. The significant difference across the four groups may therefore have been driven primarily by the association with elevated NLR rather than by a true interaction between inflammatory and nutritional status. Accordingly, the combined NLR–albumin finding should be interpreted as an exploratory descriptive observation rather than as evidence of a definitive biological or statistical interaction.

Serum albumin may reflect both nutritional status and systemic inflammatory burden, and hypoalbuminemia has been associated with impaired wound healing, increased infection risk, adverse surgical outcomes, and poor diabetic foot outcomes (18–21). However, mean albumin levels were low in both outcome groups, and a large proportion of the cohort was hypoalbuminemic; consequently, the 3.5 g/dL threshold resulted in unequal subgroup sizes. The wide confidence interval around the interaction estimate also indicates substantial uncertainty, likely related to the small number of patients in some subgroups. Larger studies are required to determine whether albumin status modifies the association between NLR and amputation-level progression.

### 4.4 Pathophysiological interpretation

Systemic inflammation plays an important role in the pathogenesis and progression of diabetic foot disease. Elevated NLR reflects an imbalance between neutrophil-mediated inflammatory activation and lymphocyte-mediated immune regulation (22). In patients with diabetes, chronic inflammation may contribute to endothelial dysfunction, microvascular impairment, reduced tissue perfusion, infection progression, and delayed wound healing, all of which may compromise stump viability after BKA and increase susceptibility to subsequent proximal amputation (1, 23, 24).

In this context, elevated NLR should be interpreted as a marker of systemic inflammatory burden rather than as evidence of a direct causal mechanism. The association observed in the present study may reflect a broader inflammatory, infectious, and physiological stress state in patients who are more vulnerable to failure of the initial BKA level and subsequent progression to AKA.

Importantly, the relationship between inflammation and amputation-level progression is likely multifactorial. Peripheral arterial disease severity, renal function, smoking, cardiovascular comorbidities, revascularization status, osteomyelitis burden, nutritional status, and wound characteristics may all influence stump healing, residual-limb viability, and the need for secondary proximal amputation. Therefore, NLR should be considered an adjunctive biomarker that may complement but not replace comprehensive clinical, infectious, vascular, and nutritional assessment.

Another possible interpretation is that elevated NLR may reflect a systemic or local severity dimension not fully captured by the retrospectively available vascular data. Although WIfI stage was assigned by the cardiovascular surgery team during routine care, individual perfusion indices were not consistently retrievable for separate analysis. Therefore, NLR may have captured part of the systemic inflammatory or vascular-infectious burden not fully represented by the available adjustment variables.

### 4.5 Quality of life implications

Major amputations are known to be associated with significant emotional distress, reduced independence, and increased socioeconomic burden, including loss of work capacity and increased healthcare expenditures (25–27). In the present study, postoperative SF-12v1 mental component scores (MCS) were significantly lower in patients who subsequently underwent amputation-level progression than in those who maintained the initial BKA level.

The postoperative SF-12v1 assessment was performed approximately three weeks after the index BKA and, in all patients who subsequently progressed to AKA, before conversion to AKA. However, stump failure, infection, tissue necrosis, or uncertainty regarding the need for further surgery may already have been present at the time of assessment. Therefore, the lower postoperative MCS should be interpreted as reflecting the ongoing adverse clinical course and associated psychological burden rather than as a predictor preceding stump deterioration or amputation-level progression.

Postoperative physical component scores (PCS) were numerically higher in patients with amputation-level progression than in those who maintained the initial BKA level (32.60 vs. 31.49), although this difference was not statistically significant. Given the small numerical difference and the generally low early postoperative PCS values in both groups, this finding should not be interpreted as evidence of better physical health among patients with progression.

Although all patients underwent a separate SF-12v1 assessment before the index BKA, the standard four-week recall period used for the postoperative week-3 assessment extended approximately one week into the period preceding the index BKA. Therefore, the postoperative scores may not have exclusively reflected postoperative health status. Despite these limitations, patients experiencing ongoing stump complications may be particularly vulnerable to early psychological distress and may benefit from timely psychological assessment, rehabilitation support, and comprehensive multidisciplinary management.

### 4.6 Clinical implications

From a clinical perspective, preoperative NLR may serve as a simple and readily available adjunctive marker of systemic inflammatory burden in patients undergoing BKA for diabetic foot disease. Recent studies have linked elevated NLR with several adverse diabetic foot outcomes, including impaired wound healing, nutritional risk, and amputation risk. Zhu et al. reported that NLR and nutritional status were associated with diabetic foot ulcer-related amputation, Zhao et al. found that perioperative NLR was associated with wound healing in Wagner grade 3–4 diabetic foot ulcers, and Afshar et al. reported that elevated NLR was associated with diabetic foot ulcer amputation (10, 14, 16). However, these studies primarily assessed wound healing, nutritional risk, or the occurrence of amputation rather than early amputation-level progression after an initial BKA. To the best of our knowledge, the present study is among the first to specifically evaluate the association between preoperative NLR and short-term progression from BKA to AKA in patients with diabetic foot disease.

NLR should not be used as a standalone predictor of amputation-level progression, as a clinical decision threshold, or as a substitute for comprehensive clinical, vascular, infectious, nutritional, and wound-based assessment. When interpreted together with established classification systems such as WIfI and Wagner, vascular status, infection control, nutritional status, and comorbidity profile, elevated NLR may provide additional information supporting closer postoperative monitoring and early multidisciplinary evaluation. However, the present findings do not establish that NLR-guided management improves clinical outcomes. Prospective multicenter studies with external validation are required before NLR can be incorporated into formal risk-assessment or clinical decision-making algorithms.

### 4.7 Limitations

This study has several limitations. First, its retrospective design introduces the possibility of selection and information bias and precludes causal inference. Second, the study was conducted at a single center and included a relatively limited number of patients, which may restrict the generalizability of the findings. Third, although a parsimonious multivariable logistic regression model was used to reduce the risk of overfitting, the sample size and number of outcome events limited the number of covariates that could be reliably included.

Residual confounding remains an important concern. Several clinically relevant variables that may influence stump healing and proximal amputation were not consistently available in the retrospective records or could not be incorporated into the analysis. WIfI stage was assigned during routine clinical care by an experienced cardiovascular surgeon using the Society for Vascular Surgery WIfI classification framework. However, the individual objective perfusion measurements underlying the ischemia component, including ankle–brachial index, toe–brachial index, toe pressure, and transcutaneous oxygen pressure, were not consistently retrievable. Consequently, these indices could not be examined separately, and incomplete characterization of lower-limb perfusion may have resulted in residual confounding related to vascular disease severity.

Medication exposures that may influence leukocyte subsets, including systemic corticosteroid use, were not consistently documented and could not be incorporated into the analysis. Although systemic corticosteroid therapy was not part of the routine management of diabetic foot complications, its use for unrelated comorbid conditions could not be reliably excluded. Other incompletely recorded or unavailable factors included renal function, smoking status, cardiovascular comorbidities, peripheral arterial disease severity, revascularization history, osteomyelitis burden, and detailed wound characteristics. These factors may affect stump healing, residual-limb viability, infection control, and the need for secondary proximal amputation. In addition, inflammatory markers such as NLR and CRP may be influenced by concurrent infection, systemic inflammation, renal dysfunction, medication exposure, and other comorbid conditions that were not fully controlled in this dataset. Elevated NLR may therefore partly reflect unmeasured overall disease severity or medication-related alterations in leukocyte counts rather than an association attributable specifically to the inflammatory marker itself.

The 3-month follow-up period was selected to capture early progression from BKA to AKA, which is more likely to reflect early stump failure, persistent or progressive infection, ischemic non-healing, or inadequate local disease control after the index amputation. However, this endpoint does not represent long-term limb-related outcomes. A longer follow-up period might have identified delayed proximal amputations associated with recurrent ulceration, new infection, prosthetic use, rehabilitation-related factors, or progression of systemic vascular disease. Death before outcome ascertainment represents a competing event for amputation-level progression. Although only one such patient was identified, the exclusion of this patient and those with insufficient 3-month follow-up may have introduced attrition bias and affected the observed progression rate. Further studies are therefore required to determine whether preoperative NLR is also associated with delayed proximal amputation and other longer-term outcomes.

The multivariable model was developed primarily to assess adjusted associations rather than to establish a clinical prediction tool. Internal validation using bootstrap resampling or cross-validation was not performed. Accordingly, model stability, optimism-corrected discrimination, and calibration could not be assessed. The ROC-derived cutoff, sensitivity, specificity, and adjusted probability estimates were derived and evaluated in the same cohort and should therefore be considered exploratory, potentially optimistic, and not suitable for individual clinical decision-making without further validation. The nonsignificant Hosmer–Lemeshow result should similarly be interpreted as indicating that lack of fit was not detected rather than as evidence of good calibration, particularly given the limited sample size. The decile-based calibration plot was also descriptive and potentially unstable because of the small number of patients within each decile.

Interobserver reliability for Wagner grade and WIfI stage was not assessed because each classification was assigned by a single experienced specialist. Although the use of designated assessors ensured consistency in routine classification, the absence of formal reliability assessment remains a limitation.

The standard SF-12v1 uses a four-week recall period. Therefore, although all patients underwent a separate assessment before the index BKA, the postoperative assessment performed at week 3 may have included approximately one week preceding the index operation and may not have exclusively reflected postoperative health status. Moreover, in patients who subsequently progressed to AKA, the postoperative assessment was performed during a period when stump failure, infection, tissue necrosis, or uncertainty regarding further surgery may already have been present. Consequently, the lower postoperative MCS should be interpreted as reflecting the ongoing clinical course and associated psychological burden rather than as a predictor preceding amputation-level progression.

Outcome ascertainment was not blinded to the exposure variables. Progression to AKA represented a clinical treatment decision rather than a purely objective event, and the treating team had access to the patients’ inflammatory markers, including NLR and CRP. It is therefore possible that markedly elevated inflammatory markers influenced the timing of conversion or lowered the clinical threshold for proceeding to a more proximal amputation. This may have introduced decision or ascertainment bias and could have contributed to an overestimation of the observed association between preoperative NLR and early amputation-level progression. This limitation is inherent to the retrospective, routine-care design of the study.

Overall, preoperative NLR remained associated with early progression from BKA to AKA after adjustment for the selected available covariates; however, residual confounding cannot be excluded. The findings should therefore be interpreted cautiously and regarded as hypothesis-generating rather than as evidence of validated predictive performance. Prospective multicenter studies with larger cohorts, longer follow-up, comprehensive vascular, renal, infectious, nutritional, and wound-related data, and appropriate internal and external validation are needed to confirm these findings and evaluate their potential clinical utility.

## 5 Conclusion

In conclusion, preoperative NLR was associated with early amputation-level progression from BKA to AKA in patients with diabetic foot disease after adjustment for selected available clinical variables. However, given the retrospective single-center design, limited sample size, and potential for residual confounding, this finding should be interpreted cautiously. NLR may serve as an inexpensive and readily available adjunctive inflammatory marker, but it should not replace comprehensive clinical, vascular, infectious, nutritional, and wound-based assessment. Prospective multicenter studies incorporating detailed vascular, renal, infectious, nutritional, wound-related, and comorbidity-related variables are needed to confirm these findings and further clarify the association between NLR and amputation-level progression.

## Data Availability

The raw data supporting the conclusions of this article will be made available by the authors, without undue reservation.

## Supplementary material

Supplementary Figure 1 **STROBE flow diagram of patient selection.** A total of 144 consecutive adult patients undergoing primary transtibial amputation for diabetic foot complications were screened for eligibility. After the exclusion of 39 patients, 105 patients were included in the final analytic cohort. At 3 months, 52 patients had maintained the initial below-knee amputation level, whereas 53 had progressed to above-knee amputation. BKA, below-knee amputation; AKA, above-knee amputation.
